# Outcomes and Safety of Suprapubic vs Urethral Catheterization Following Pelvic Fascia‒Sparing Robotic Prostatectomy

**DOI:** 10.1097/UPJ.0000000000000492

**Published:** 2023-12-05

**Authors:** Samuel Engelsgjerd, Sarah Kodres-O'Brien, Eshrar Choudhury, Belén Mora Garijo, J. Bradley Mason, Keith J. Kowalczyk

**Affiliations:** 1 Department of Urology, MedStar Georgetown University Hospital, Washington, District of Columbia; 2 Georgetown University School of Medicine, Washington, District of Columbia

**Keywords:** pelvic fascia sparing, robotic surgery, prostatectomy, suprapubic catheter, urethral catheter

## Abstract

**Introduction:**

Urethral catheter (UC) discomfort remains a burden following robotic-assisted radical prostatectomy (RARP). Suprapubic catheters (SPCs) may reduce patient discomfort and increase satisfaction. Pelvic fascia‒sparing (PFS) RARP reduces the technical challenges of intraoperative SPC placement. We examined postoperative outcomes of SPC vs UC placement following PFS-RARP.

**Methods:**

We conducted a retrospective review of a prospective institutional review board‒approved database of PFS-RARP patients from June 2020 to December 2022 receiving SPC (n = 108) or UC (n = 104) postoperatively. Demographics and clinical and perioperative outcomes were captured. Postoperative patient-reported quality of life was measured using EPIC-CP (Expanded Prostate Cancer Index Composite for Clinical Practice). Patients with intraoperative complications or intraoperative leaks or undergoing salvage prostatectomy were excluded. Univariate and multivariate regression analyses were performed to compare outcomes.

**Results:**

No significant differences in demographics or oncologic outcomes existed. There were no differences in complications, including urethral stricture or anastomotic leak. Men receiving SPC vs UC had earlier return to continence (7 vs 16 days, *P* < .001) and higher continence rates at catheter removal (67.6% vs 43.3%, *P* = .0003). On adjusted analyses, SPC was an independent predictor of continence at catheter removal (OR 2.21, *P* = .023). There were no differences between groups in preoperative or postoperative EPIC-CP scores, including no differences in postoperative quality of life (*P* = .46).

**Conclusions:**

SPC after PFS-RARP is a safe and feasible alternative to UC. SPC is associated with an earlier return to continence and higher continence rates at catheter removal. Use of SPC may increase overall patient satisfaction following PFS-RARP.

While robotic-assisted radical prostatectomy (RARP) techniques have improved postoperative pain and hastened patient recovery, the need for a bothersome postoperative urethral catheter (UC) continues to cause discomfort for patients.^1-3^ UCs ensure complete bladder drainage and possibly facilitate proper anastomotic healing at the surgical site.^4,5^ However, UCs often results in irritative symptoms, including general pain/discomfort, blood in the urine, and involuntary bladder spasms known as catheter-related bladder discomfort (CRBD).^6,7^

Suprapubic catheter (SPC) insertion provides a more comfortable alternative to urethral catheterization following RARP. Previous studies investigating SPC use after standard RARP have shown several benefits, including improved penile pain scores, decreased need for anticholinergic medications to manage CRBD-associated spasms, and enhanced overall patient satisfaction in individuals managed with SPC.^8,9^ While the placement of SPC following conventional prostatectomy can pose technical challenges, the technique of pelvic fascia‒sparing (PFS) RARP overcomes these difficulties by preserving the bladder in its anatomical position. However, there is limited data available regarding the impact of SPC utilization on quality of life (QoL) and functional outcomes following PFS-RARP.^10^ Moreover, there are still concerns regarding the safety of routinely employing SPC vs UC, particularly with respect to bladder neck contracture and urine leakage.

The aim of this study is to investigate the use of SPC in PFS-RARP. The hypothesis is that SPC placement is both safe and uncomplicated, without increasing the risk of postoperative anastomotic stricture or leak. Additionally, it is posited that SPC utilization may potentially improve QoL compared to PFS-RARP with postoperative UC drainage.

## Materials and Methods

We retrospectively reviewed a prospectively maintained, institutional review board‒approved database of PFS-RARP cases performed by a single surgeon (K.J.K.) at MedStar Georgetown University Hospital from June 2020 to December 2022. Patients were categorized postoperatively into 2 groups: those who received an SPC and those who received a UC. The study included a total of 212 patients; 104 patients received a UC and 108 received an SPC. Comprehensive data including race (African American, Asian, Caucasian, Middle Eastern, and Hispanic), BMI, Charlson Comorbidity Index, Gleason Grade Group, PSA levels, clinical and pathologic staging, prostate weight, and continence status at the time of catheter removal were prospectively captured and recorded in the database

**Figure 1.**
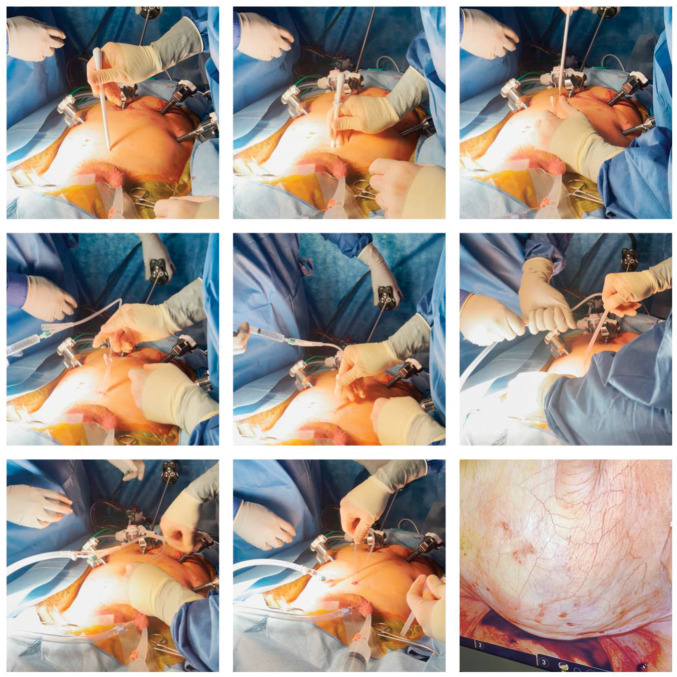
Photographs of intraoperative suprapubic catheter placement using Supra-Foley Suprapubic Catheter Introducer to insert a 14F suprapubic catheter.

SPCs were inserted under direct laparoscopic visualization following the procedure in patients exhibiting a negative leak test (minimum of 120 cc sterile fluid) while they were still positioned in steep Trendelenburg. Patients who showed positive intraoperative leak tests, as well as men undergoing salvage PFS-RARP, were excluded from this study. We utilized both the O’Brien SPC Kit and the Supra-Foley Suprapubic Catheter Introducer to insert a 14F SPC. However, we have recently developed a preference for the latter due to its ease of use and decreased time of placement (Figure 1). Catheters were left in place for 7 days postoperatively in both cohorts.

Preoperative and 6-week postoperative Expanded Prostate Cancer Index Composite for Clinical Practice (EPIC-CP) scores were collected to evaluate symptoms of urinary incontinence, urinary irritability/obstruction, vitality/hormone, and total QoL. Time to continence, as measured by both zero and 1 safety pad usage, was assessed. Oncologic indicators, including positive surgical margins (both focal and nonfocal), and the presence or absence of margins were also evaluated.

Univariate outcomes were analyzed using the χ^2^ test and ANOVA where appropriate, considering a *P* value of less than .05 as statistically significant. Categorical variables are represented by the mean and standard deviation, whereas continuous variables are depicted by median and interquartile range. An exception was made for the number of pads used per day, as medians did not vary significantly due to low fluctuation.

To control for confounding variables and follow-up disparities, we employed multivariate logistic regression to evaluate variables predicting continence at catheter removal. Furthermore, Kaplan-Meier curves were constructed to compare the time to continence.

## Results

Table 1 summarizes the baseline demographic and outcome data of both cohorts. There were no significant differences in age, race, or BMI between both cohorts. Additionally, the median total PSA and Gleason Grade Group were nearly identical between the 2 groups. There were 3 (2.78%) postoperative complications in the SPC cohort (Clavien-Dindo scores of 1, 2, and 3b) compared to 4 (3.85%) in the UC cohort (Clavien-Dindo scores of 2, 3a, 3a, and 3b). There were no symptomatic bladder neck contractures or urethral strictures in patients in either cohort. No complications in the SPC cohort were catheter related. The average follow-up time was 14 months for the SPC group and 44 months for the UC group. Average console time was slightly longer in the UC group at 122.4 minutes compared to 113.0 minutes in the SPC group (*P* = .006), and average estimated blood loss was also slightly higher in the UC group at 163 mL compared to 108 mL in the SPC group (*P* = .001). There were no differences in positive surgical margin or length of stay between the 2 groups.

**Table 1.** Differences in Demographic and Outcome Variables Between Men Receiving Suprapubic Catheter vs Urethral Catheter

	SPC (N = 108)	UC (N = 104)	*P* value
Age, mean ± SD, y	64 ± 6.5	62 ± 6.6	**< .01**
Race, No. (%)			.16
Asian	4 (3.70)	6 (5.77)	
African American	33 (30.56)	39 (37.50)	
Caucasian	65 (60.19)	51 (49.04)	
Middle Eastern	3 (2.78)	2 (1.92)	
Hispanic	3 (2.78)	6 (5.77)	
BMI, mean ± SD	28.62 ± 4.6	28.25 ± 4.3	.55
Total PSA, median	7	6.8	.06
Gleason Grade Group, median	2	2	.92
Postoperative complications, No. (%)	3 (2.78)	4 (3.85)	.67
Clavien-Dindo grade I	1 (0.93)	0	
Clavien-Dindo grade II	1 (0.93)	1 (0.96)	
Clavien-Dindo grade IIIa	0	2 (1.92)	
Clavien-Dindo grade IIIb	1 (0.93)	1 (0.96)	
Console time, mean ± SD, min	113.0 ± 24.7	122.4 ± 24.7	**< .01**
Estimated blood loss, mean, mL	108	163	**< .01**
Positive margin, No. (%)	37 (34.26)	30 (28.85)	.40
Focal margin	25 (23.15)	20 (19.23)	
Nonfocal margin	12 (11.11)	10 (9.62)	
LOS, mean ± SD, d	1.04 ± 0.38	1.09 ± 0.37	.34
Baseline EPIC-CP, mean ± SD	7.82 ± 7.23	8.06 ± 6.98	.82
EPIC-CP UI	1.00 ± 1.52	1.00 ± 1.69	.44
EPIC-CP vitality	1.41 ± 2.33	1.50 ± 2.23	.80
6-wk EPIC-CP, mean ± SD	16.08 ± 7.58	15.83 ± 7.05	.82
EPIC-CP UI	3.10 ± 2.46	3.18 ± 2.38	.83
EPIC-CP vitality	1.33 ± 2.11	1.57 ± 2.31	.46
Time to continence (0-1 pads), median, d	7	16	**< .01**
Continence at catheter removal, No. (%)	73 (67.6)	45 (43.3)	**< .01**
Current No. pads, mean ± SD	0.39 ± 0.65	0.25 ± 0.52	.08

Abbreviations: EPIC-CP, Expanded Prostate Cancer Index Composite for Clinical Practice; LOS, length of stay; SPC, suprapubic catheter; UC, urethral catheter; UI, urinary incontinence.

Bolded text indicates statistically significant data (*P* < .01).

**Figure 2.**
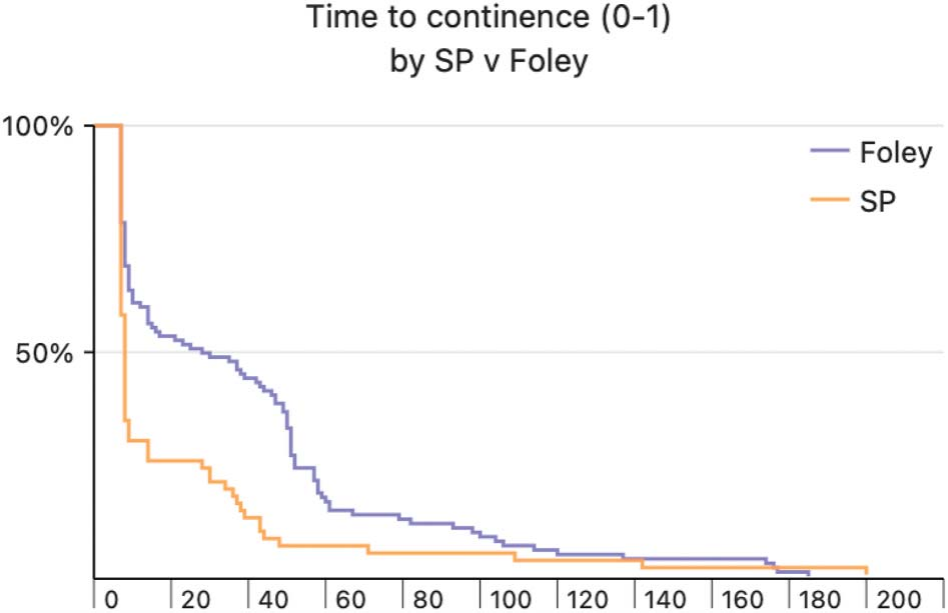
Log-rank test outcomes for continence (0-1 pad definition) between men receiving suprapubic (SP) vs urethral Foley catheter (*P* < .001).

SPC patients had a faster return to continence with a median time to continence of 7 days compared to the UC patients with a median time to continence of 16 days (*P* < .001), and more SPC patients were continent at the time of catheter removal (67.6% vs 43.3%, *P* < .001). Figure 2 shows a Kaplan-Meier curve for time to continence between groups (defined as 0-1 pads per day), showing a faster return to continence with SPC vs UC. Multivariate logistic regression analyzing variables associated with continence at catheter removal (defined as 0-1 pads) was performed (Table 2) and showed that SPC utilization was significantly associated with being continent at catheter removal compared to UC (OR 2.21, 95% CI 1.12-4.38, *P* = .023).

**Table 2.** Multivariate Logistic Regression Analyzing Variables Associated With Continence at Catheter Removal (Defined as 0-1 Pads)

	Odds ratio (95% CI)	*P* value
Age, median (IQR), y	0.98 (0.93, 1.04)	.558
Race (grouped), median (IQR)		
Caucasian	1.13 (0.54, 2.35)	.747
Other	1.90 (0.56, 6.39)	.301
SPC vs UC, median (IQR)	2.21 (1.12, 4.38)	.023
pT3 vs pT2, median (IQR)	0.66 (0.31, 1.38)	.267
Pre-EPIC UI, median (IQR)	0.82 (0.65, 1.04)	.098

Abbreviations: EPIC, Expanded Prostate Cancer Index Composite; SPC, suprapubic catheter; UC, urethral catheter; UI, urinary incontinence.

**Figure 3.**
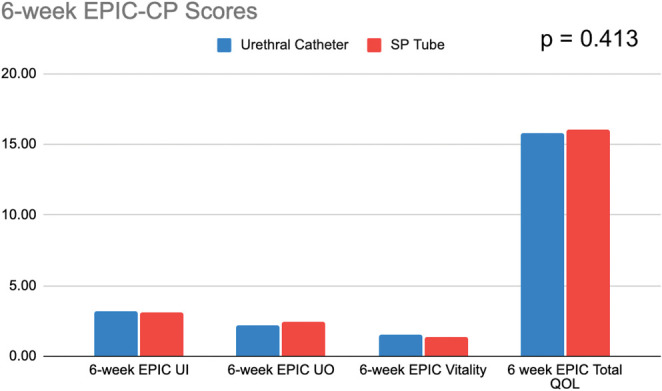
Six-week Expanded Prostate Cancer Index Composite (EPIC) for Clinical Practice (CP) scores for men receiving suprapubic (SP) vs urethral Foley catheter. QOL indicates quality of life; UI, urinary incontinence; UO, urinary obstruction.

Both groups had similar baseline EPIC-CP scores preoperatively and postoperatively, further broken down by questionnaire section and shown in Figure 3. There were no significant differences in the average current number of pads between SPC and UC (0.39 vs 0.25, *P* = .08).

## Discussion

UC is associated with a wide range of bothersome urinary symptoms, including penile pain and CRBD.^6,7^ Studies have demonstrated that when compared to UC, patients managed with SPC have improved penile pain scores, decreased use of anticholinergic medications indicating less bothersome CRBD-associated spasms, and improved QoL.^8,9^ However, there is limited evidence on the use of SPC as an alternative to UC in patients undergoing PFS-RARP.^11^ Galfano et al have the only other similar study in a cohort of men undergoing PFS-RARP, and found decreased pain in men receiving SPC vs UC following surgery, with no difference in complications.^11^ Our study further validates the safety of SPC placement following PFS-RARP and is the first to investigate functional outcomes and patient-reported QoL using the EPIC-CP score. We hypothesized that patients managed with SPC would have improved QoL without compromised functional outcomes, and that this would demonstrate that SPC is a promising and safe alternative to UC in these patients.

Our study has many important findings. First, we hypothesized that there would be no functional differences between groups. However, the SPC group had a much earlier return to continence compared to the UC group with a median time to continence of 7 vs 16 days, respectively (*P* < .001). Additionally, continence at catheter removal was significantly higher in the SPC group vs the UC group (67.6% vs 43.3%, *P* < .001). We propose that the observed difference in continence outcomes between patients managed with SPC and those managed with UC is more likely due to patient confidence in achieving continence and their ability to perform early Kegel exercises with SPC, rather than any anatomical or physiological variations. In the case of patients managed with SPC, the catheter is capped on the evening prior to removal during a clinic visit, allowing the patient to undergo a void trial in the comfort of their own homes. This enables them to gain confidence in their ability to void spontaneously before catheter removal. In contrast, patients managed with UCs do not have the opportunity to undergo a void trial before catheter removal, which may contribute to their delayed continence. We believe that the delayed continence in UC patients is not solely due to the physical presence of the catheter but rather related to the lack of confidence. Another potential contributing factor to earlier continence in the SPC group is their ability to engage in Kegel exercises earlier without the discomfort caused by a UC. Further, surgeon learning curve and experience likely also contributes to the improved continence we observed. This finding is novel, as a previous study by Galfano et al^11^ did not identify any differences in continence outcomes between the 2 catheter management approaches.

Second, there are no significant differences between groups regarding the current number of safety pads being used, with the SPC group currently using 0.39 pads and the UC group using 0.25 pads (*P* = .08). This is despite the UC group having an overall longer time since surgery as we converted to almost exclusively managing these patients with SPC, and therefore might expect the SPC group to be using more pads as they continue to recover continence. However, we see this is not the case, and this further supports the assertion that there are no significant functional harms related to the use of SPC as an alternative to UC, and there could potentially be some functional benefits early after surgery. Greater numbers of patients are needed to determine if these functional benefits early on are true and to determine the mechanism leading to an earlier return of continence in the SPC group.

Third, while previous studies have shown improvements in QoL for patients managed with SPC vs UC, our study did not find significant differences in patient-reported QoL between groups. The SPC group had a slightly lower EPIC-CP vitality score at the 6-week follow-up (1.33) than the UC group (1.57), where a lower score indicates fewer bothersome symptoms and therefore greater vitality, but this difference was not statistically significant (*P* = .46). There was also no difference in total EPIC-CP scores at 6 weeks, indicating no difference in overall bothersome symptoms including urinary, sexual, and vitality-related symptoms. It is important to note that the EPIC-CP questionnaire was filled out at the 6-week postoperative appointment, which is long after catheter removal. To truly study QoL differences between the SPC and UC groups related to the catheters themselves, a questionnaire should be used while the catheter is in place.

The majority of differences in QoL in previous studies have been posited to be due to differences in penile pain associated with UC and greater frequency of bladder spasms.^8,9^ One meta-analysis found a significant improvement in catheter-related bother in the SPC group vs the UC group on postoperative day 7.^11^ It is possible that our study does not show significant differences in QoL outcomes between groups because the questionnaire is not administered when the catheters are in place, but rather several weeks after bothersome catheter-associated symptoms have resolved. More research is needed to determine if there is a difference in QoL between patients managed with SPC vs UC following PFS-RARP. Furthermore, while the EPIC-CP questionnaire assesses vitality, it does not specifically assess penile pain scores, which account for a large portion of the bother associated with UC. Future research should aim to study specific catheter-related symptoms, such as penile pain, bladder spasms, use of anticholinergic medications, and overall satisfaction as it relates to the catheter.

Fourth, in addition to bladder decompression, UC has been utilized to promote proper anastomotic healing following RARP.^5^ Numerous studies have shown that rates of acute urinary retention are higher with earlier removal of UC, suggesting that the catheter aids in the patency of the anastomosis shortly after surgery and allows swelling to subside before its delayed removal.^12-14^ A concern with SPC is that the lack of a catheter traversing the anastomosis may interfere with the proper anastomotic healing and increase rates of postoperative acute urinary retention, bladder neck contracture, or anastomotic stricture. It is therefore important to note that in our study rates of complications were low in both the SPC and UC groups (2.78% and 3.85%, *P* = .67). Additionally, no patients in either cohort had a bladder neck contracture or stricture with an average follow-up of 14 months for the SPC group and 44 months for the UC group. There was no incidence of postoperative urinary retention or hypercontinence in the SPC group, whereas 3 patients with UCs were excluded from the study due to postoperative retention. This may be an additional benefit of SPC over UC as the void trial enables the anticipation and avoidance of postoperative urinary retention requiring potential traumatic replacement of an indwelling UC. This shows that SPC is safe to do after PFS-RARP without increasing rates of complications or delaying healing.

Finally, it is worth mentioning that SPC placement following RARP has been done, and its safety and efficacy studied before, but the literature in the setting of PFS-RARP is lacking. In 2013, Ghani et al published a step-by-step guide for the placement of an SPC following RARP.^15^ They noted in their guide the significant technical difficulties of placing an SPC after having just dissected the anterior pelvic fascia and dropping the bladder away from the anterior abdominal wall. One of the steps in the guide involves pulling the bladder back toward the anterior surface. However, with PFS-RARP, the detrusor apron and pre-pelvic fascia are left intact, avoiding this complicated step and making SPC placement technically easier and quicker. The statistically significant shorter operating time and lower estimated blood loss are likely byproducts of the inherent bias in a single-surgeon study where these findings were related to surgeon experience rather than UC vs SPC.

There are several limitations to this study. First, our questionnaire is limited in the specific questions we are able to answer, and it is possible the significance of the data is affected by the timing of questionnaire administration. In the future, it would be helpful to add questions related to penile or suprapubic pain while having the catheter in place. As mentioned, the 6-week administration of the EPIC-CP is well after catheter removal, and thus vitality scores from that time are unlikely to reflect vitality directly associated with the catheter. Second, this is a retrospective review of a prospective database and not a randomized controlled trial. Finally, as a single-surgeon series, where UC was used for approximately the first 100 patients and SPC was used for the latter patients, it is possible that increasing surgeon experience introduces bias into the results.

## Conclusions

Our study provides compelling evidence supporting the use of SPC as a safe and viable alternative to UC after PFS-RARP. We observed no significant differences in complications between the 2 groups, including the absence of strictures or bladder neck contractures. Importantly, none of the patients in the SPC group experienced postoperative urinary retention, highlighting the additional benefit of the SPC void trial, which is not feasible with UC. Furthermore, there was a substantial disparity in time to continence, with the SPC group achieving continence significantly earlier and demonstrating higher rates of continence at the time of catheter removal compared to the UC group. These findings provide strong support for the safe and effective use of SPC instead of UC following PFS-RARP.

## References

[1] Bill-Axelson A Holmberg L Garmo H . Radical prostatectomy or watchful waiting in early prostate cancer. N Engl J Med. 2014;370(10):932-942. 24597866 10.1056/NEJMoa1311593 PMC4118145

[2] Yao XD Liu XJ Zhang SL Dai B Zhang HL Ye DW. Perioperative complications of radical retropubic prostatectomy in patients with locally advanced prostate cancer: a comparison with clinically localized prostate cancer. Asian J Androl. 2013;15(2):241-245. 23223030 10.1038/aja.2012.120 PMC3739142

[3] Veerman H Houwink API Schutte PFE . Intraoperative strategies to reduce catheter-related bladder discomfort in the early postoperative period after robot-assisted radical prostatectomy. J Urol. 2021;205(6):1671-1680. 33605794 10.1097/JU.0000000000001645

[4] Shelfo SW Obek C Soloway MS. Update on bladder neck preservation during radical retropubic prostatectomy: impact on pathologic outcome, anastomotic strictures, and continence. Urology. 1998;51(1):73-78. 10.1016/s0090-4295(97)00463-9 9457292

[5] Steiner MS Morton RA Walsh PC. Impact of anatomical radical prostatectomy on urinary continence. J Urol. 1991;145(3):512-515. 1997701 10.1016/s0022-5347(17)38382-9

[6] Saint S Trautner BW Fowler KE . A multicenter study of patient-reported infectious and noninfectious complications associated with indwelling urethral catheters. JAMA Intern Med. 2018;178(8):1078-1085. 29971436 10.1001/jamainternmed.2018.2417 PMC6143107

[7] Binhas M Motamed C Hawajri N Yiou R Marty J. Predictors of catheter-related bladder discomfort in the post-anaesthesia care unit. Ann Fr Anesth Reanim. 2011;30(2):122-125. 21277735 10.1016/j.annfar.2010.12.009

[8] Morgan MS Ozayar A Friedlander JI . An assessment of patient comfort and morbidity after robot-assisted radical prostatectomy with suprapubic tube versus urethral catheter drainage. J Endourol. 2016;30(3):300-305. 26472083 10.1089/end.2015.0206

[9] Krane LS Bhandari M Peabody JO Menon M. Impact of percutaneous suprapubic tube drainage on patient discomfort after radical prostatectomy. Eur Urol. 2009;56(2):325-330. 19394131 10.1016/j.eururo.2009.04.018

[10] Egan J Marhamati S Carvalho FLF . Retzius-sparing robot-assisted radical prostatectomy leads to durable improvement in urinary function and quality of life versus standard robot-assisted radical prostatectomy without compromise on oncologic efficacy: single-surgeon series and step-by-step guide. Eur Urol. 2021;79(6):839-857. 32536488 10.1016/j.eururo.2020.05.010

[11] Galfano A Secco S Panarello D . Pain and discomfort after Retzius-sparing robot-assisted radical prostatectomy: a comparative study between suprapubic cystostomy and urethral catheter as urinary drainage. Minerva Urol Nefrol. 2019;71(4):381-385. 31144484 10.23736/S0393-2249.19.03237-5

[12] Jian Z Feng S Chen Y . Suprapubic tube versus urethral catheter drainage after robot-assisted radical prostatectomy: a systematic review and meta-analysis. BMC Urol. 2018;18(1):1. 29304797 10.1186/s12894-017-0312-5 PMC5756422

[13] Khemees TA Novak R Abaza R. Risk and prevention of acute urinary retention after robotic prostatectomy. J Urol. 2013;189(4):1432-1436. 23021999 10.1016/j.juro.2012.09.097

[14] Patel R Lepor H. Removal of urinary catheter on postoperative day 3 or 4 after radical retropubic prostatectomy. Urology. 2003;61(1):156-160. 12559288 10.1016/s0090-4295(02)02105-2

[15] Ghani KR Trinh QD Sammon JD . Percutaneous suprapubic tube bladder drainage after robot-assisted radical prostatectomy: a step-by-step guide. BJU Int. 2013;112(5):703-705. 23924427 10.1111/bju.12071

